# The effects of training on hormonal concentrations and physical performance of football referees

**DOI:** 10.14814/phy2.14740

**Published:** 2021-05-01

**Authors:** Antonella Muscella, Erika Stefàno, Santo Marsigliante

**Affiliations:** ^1^ Department of Biological and Environmental Science and Technologies (Di.S.Te.B.A University of Salento Lecce Italy

**Keywords:** cortisol, football, maximal oxygen consumption, testosterone, training, Yo–Yo intermittent recovery test level 1 (YYIRT1)

## Abstract

As no study has explored the impact of physical stress on hypothalamic‐pituitary‐gonadal axis hormones over a long period, the purpose of this study was to determine the effects of the football season period on plasma cortisol and testosterone concentrations and referee's physical performances. Physical tests and plasma cortisol and testosterone concentrations were assayed before the beginning of the training period, just after the training period, at the middle of the season, and at the end of the season, in 29 male football referees and 30 healthy control subjects. Results showed significant differences in hormone concentrations at the four‐time points evaluated. Plasma cortisol increased during the first training period from 15.8 ± 3.8 to 21.7 ± 5.1 µg/dl (*p* < 0.001), then decreased during the season and at the end of it was 18.7 ± 2.4 µg/dl. Before the beginning of the training period, plasma testosterone concentration was 386.1 ± 58.8 ng/dl; after the training period, it increased to 572.2 ± 88.1 ng/dl (*p* < 0.001) and then returned to baseline levels at the end of the season. Between the start of the training period and the end of the season, significant differences were observed in physical performances of referees. Plasma cortisol and testosterone levels significantly (*p* < 0.0001 for both) correlated with Yo‐Yo intermittent recovery test level 1 (YYIRT1) and maximal oxygen consumption (VO_2max_) at the end of the training period. In the middle season, plasma testosterone concentration only significantly (*p* < 0.0001) correlated with YYIRT1 and VO_2max_. These data underline the importance of set up training protocols that present the prospective to favor positive physiological adaptations.

## Graphical abstract

Referees’ physical preparation improved their physical performances, assed by Yo‐Yo intermittent recovery test level 1 (YYIRT1), and running speed test. Significant differences in cortisol and testosterone levels at the four‐time points were observed. Plasma cortisol and testosterone levels significantly correlated with YYIRT1 and maximal oxygen consumption during the training period. The results underscore the importance of establishing training protocols to promote referee's positive physiological adaptations.

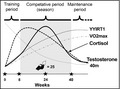

**Abbreviations**
VO_2max_: maximal oxygen consumption
YYIRT1: Yo‐Yo intermittent recovery test level 1

## 1 INTRODUCTION

Football is the most popular sport in the world (Keen, 2018) and, from the point of view of training and physical performance, attention is mainly focused on players. However, soccer referees also play an important role in applying the rules of the game by closely observing the players. It is important that referees pay attention to all players’ movements in order to sanction the violation of the rules and to prevent foul play injuries more effectively. From this, it follows that also the referees must train in order to keep up with the game at all times obtaining optimal positioning in order to make their critical decisions (Mallo et al., 2012). During a match football referees cover about 10–12 km (Krustrup & Bangsbo, 2001; Weston et al., 2007) performing up to 1269 changes in activity; they repeatedly reach 85 to 95% of their maximum heart rate (Weston et al., 2012). The referees’ high‐speed running distances have been observed to decrease during the later stages of the match (Krustrup & Bangsbo, 2001); this may be due to accumulated fatigue (Krustrup et al., 2009) provoked by the high demands of physical and physiological correspondence (Mallo et al., 2012), as demonstrated by the increase in lactate in the blood and by the performance of the sprint decrease (Castillo et al., 2016). Currently, the fatigue of football referees during the season has increased due to the greater number of matches at all competitive levels (Da Silva et al., 2011). Therefore, referees are subjected to physical and psychological stress, due to complex decision making by dealing with players, coaches, and audiences. Physical training affects the state of the hypothalamic–pituitary–gonadal axis hormones (Hackney et al., 2003) and this is particularly true for high intensity and physically demanding sports (Aldous et al., 2016; Hammami et al., 2017). Cortisol plays a key role in the regulation of physical and mental stress regulating most of the catabolic adaptations to exercise. Plasma cortisol concentrations depend upon the intensity and duration of the exercise as well as on physical fitness (Dickerson & Kemeny, 2004). As regards testosterone, it improves athletic performance and has important roles in muscle hypertrophy and in the synthesis of muscle glycogen (Wood & Stanton, 2012).

Thus, testosterone and cortisol values can be considered important parameters that help to evaluate the influence of training and competition as a consequence of the balance between anabolic and catabolic processes (Urhausen et al., 1995). Despite some reports showing seasonal adaptation in physical performances of football referees (Weston et al., 2004, 2011), no study has explored the impact of the above‐mentioned physical stress on cortisol or testosterone over a long period. For these reasons, the purpose of this study was to analyze the effects of the football season period on football referee's physical performance variables and on plasma testosterone and cortisol concentrations. These new data underscore the importance of establishing training protocols that present the potential to promote referee's positive physiological adaptations.

## 2 METHODS

### 2.1 Procedures

**FIGURE 1:**
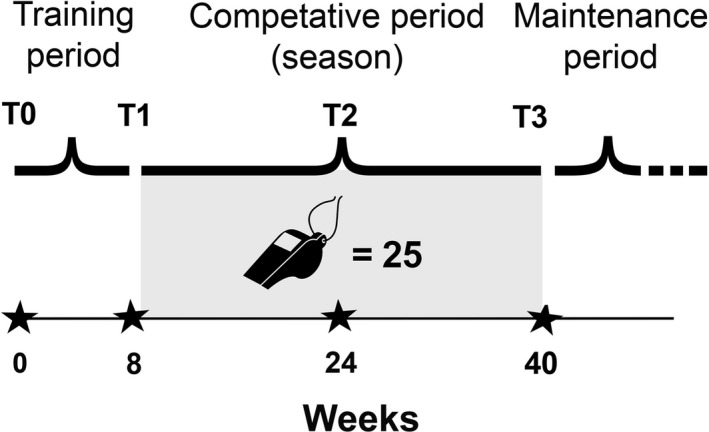
Organization of the research study protocol applied during a football season (40 weeks). Testing time‐points were four (indicated by the stars): before the beginning of the training period (T0, in September, week 0), just after the training period (T1, 8 weeks from T0), at the middle of the season (T2, 24 weeks after T0), and at the end of the season (T3, 40 weeks after T0). The number of match's officiated (25) is shown next to the whistle logo

This study was designed to analyze the effects of a 40‐week physical preparation period on cortisol concentration and physical performance in 29 football referees. The results of relevant fitness tests evaluating linear sprint, change in direction and intermittent high‐intensity performance in football referees during the competitive season 2017/2018, were also assessed. Fitness and biological tests were performed before the beginning of the training period (T0, in September), just after the training period (T1), at the middle of the season (T2), and at the end of the season (T3, in June), as reported in Figure 1. The blood samples were collected at 7:30 a.m., in the fasting state.

### 2.2 Participants

Twenty‐nine male football referees that officiated official football matches during the 2017/2018 season volunteered to participate in this study. The age of referees was in the range of 18–23 years. According to the 2016 FIFA Fitness Tests document, the referees who participated in this study were refereeing at category 1 (referees who officiate in the professional competitions organized by a professional league) and category 2 (referees who officiate in the semi‐professional and amateur competitions organized at the national level).

A control group composed of 30 non‐athletic men, of the same ages (range 18–24 years), was recruited volunteered. The control group did not receive any specific exercise protocol and was allowed to continue their training routine (4 ± 2 sessions per week). During the study, subjects were instructed to maintain their normal training diet and ingest only water in the 60 min before data collection. None of them smoked or had a significant medical or health history. Both referees and non‐athletic men took no medication before or during this study, nor were they taking any supplements or corticoids. The study was approved by the Institutional Review Board (I.R.B.) and all experiments were conducted in accordance with the 2013 Helsinki declaration and its later amendments. Written informed consent was obtained from each participant after a full explanation of the purpose and nature of all procedures used.

### 2.3 Anthropometric characteristics

Body weight and height were obtained with standard techniques to the nearest 0.1 kg and 0.1 cm, respectively, for each subject. The body fat percentage was calculated from four skins fold measurements using a Harpenden caliper (HaB International Ltd.) on the right side of the body (Jackson & Pollock, 1978). Resting Heart Rate (HR) was measured by theolar S710 heart rate monitor and data were processed by specific software. Maximal and minimal basal arterial pressures (AP) were determined by the PIC‐BS400 digital pressure. All measurements were taken at 7.30 a.m. by the same investigator, for all time periods. Anthropometric characteristics of referees and controls determined before the beginning of the training period (T0) and at the end of the season (T3) periods (mean ± *SD*), are shown in Table 1.

**TABLE 1:** Pre‐training (T0) versus the end of the season (T3) anthropometric data (body mass index, height, weight, and % body fat) of football referees and control subjects

Physical characteristics	Referees		Control subjects	
	Pre	Post	Pre	Post
Age (years)	22.5 ± 3.1	22.6 ± 4.3	21.9 ± 3.5	22.6 ± 4.3
Heigh (cm)	176.5 ± 4.2	177.7 ± 4.8	177.2 ± 3.2	177.9 ± 8.6
BMI (Kg/m^2^)	23.8 ± 0.8	21.52 ± 0.7^†^, ^§^	23.6 ± 1.4	23.1 ± 0.9
Weigh (kg)	70.5 ± 3.1	71.8 ± 5.5^§^	69.9 ± 5.1	72.2 ± 4.5
%Body fat	13.6 ± 0.7	11.2 ± 1.3^†^, ^§^	14.2 ± 1.1	13.2 ± 0.9
40‐m sprint (s)	5.9 ± 0.4	5.2 ± 0.2^†^, ^§^	7.43 ± 0.83	7.5 ± 0.8
YYIRT1 (m)	1732.3 ± 105	1925.5 ± 212^†^, ^§^	1370.2 ± 120	1422.2 ± 346
VO_2max_ (ml/Kg/min)	50.8 ± 0.8	52.7 ± 0.7^†^, ^§^	47.9 ± 1.0	48.3 ± 2.1†
Resting HR (beats/min)	66 ± 2	61 ± 3^†^	68 ± 4	68 ± 3
Maximal AP (mmHg)	108 ± 6	109 ± 6	110 ± 3	112 ± 5
Minimal AP (mmHg)	73 ± 2	72 ± 4	75 ± 2	77 ± 3

Data are presented as means (±*SD*).

† *p* < 0.05 Significantly different from T0 to T3.

§ *p* < 0.05 Significantly different from control subjects.

### 2.4 Training programs

All football referees, when healthy, attended a training program as previously reported (Muscella et al., 2020). The program consisted at least 3 training sessions per week; during the season they performed: 85 sessions of aerobic‐type training (resistance), 65 sessions of anaerobic alactacid type training (speed), 93 sessions of anaerobic lactacid type training (resistance to the speed) and 40 sessions for the improvement of the muscular strength. All referees were regularly trained 120–150 min per session, one session per day, and played on average one competitive match per week.

Thus, football referees have been involved in officiating 25 matches during the year. Physical loads during training sessions and the match were quantified using a global positioning sensor (GPS) watch (Timex Ironman Global Training, USA).

During training, football referees reach average heart rate values (HR) of 163 ± 4 beats/min that correspond to approximately 85–90% of the maximum heart rate (HR_max_). In fact, the training program, consisting mainly of intermittent running at heart rates above 90% HR_max_. Each training session consisted of a 10 min warm‐up followed by either long‐duration running intervals (4 × 4 min or 8 × 2 min) or short duration running intervals (16 × 1 min or 24 × 30 s) with a 2:1 ratio between exercise and rest. Since nutritional guidelines were provided to all referees, they followed the same nutritional and hydration protocol during the period considered (40 weeks).

### 2.5 Physical fitness characteristics

To get information regarding the physiological status of football referees, we have used tests that have been frequently used as part of their match selection criteria: Yo–Yo intermittent recovery test level 1 (YYIRT1) and running speed test. The YYIRT1 was used as a predictor of high‐intensity aerobic capacity and VO_2max_ (Hammami et al., 2017; Krustrup & Bangsbo, 2001; Krustrup et al., 2003) and, before data collection, all participants underwent the familiarization of tests. Each participant continues running between two parallel lines 20 meters apart, at progressively increasing speeds controlled by the “beeps” on a CD. The subjects had a 10 second active rest period (decelerating and walking back to the starting line) between each running bout. YYIRTL1 was also used to estimate VO_2max_ (ml·min^−1^·Kg^−1^), by the equation of Bangsbo (Krustrup & Bangsbo, 2001). In the running speed test, the participants performed three maximal 40‐m sprints, measured with an infrared photoelectric cell (Speed‐trap II Wireless Timing System; Brower Timing System, Draper, UT), as previously described (Muscella et al., 2019).

### 2.6 Blood analysis

To reduce the effects of diurnal variation on hormonal concentrations, blood samples were drawn always at 8:00 in the morning. After centrifugation, the serum was removed and frozen at −20°C for later analysis. As previously reported (Muscella et al., 2019), serum testosterone and cortisol were analyzed by IMMULITE 2000 Immunoassay System (Medical Systems). Intra‐ and inter‐assay coefficients of variance for cortisol were 4.6% and 7.6%. The intra‐ and inter‐assay coefficients of variance for testosterone were 3.7% and 5.6%. Serum testosterone and cortisol reference ranges were 10–75 ng/dl and 7–25 g/dl, respectively.

### 2.7 Statistic analysis

Data, collected in a blinded fashion, were analyzed by GraphPad PRISM 5 software (GraphPad Software). All variables used in the study were checked for the normality of distribution before the analyses (Kolmogorov‐Smirnov tests). Results are expressed as means ± standard deviations (*SD*). Student's unpaired *t*‐test and Kolmogorov‐Smirnov tests were used. The statistical Friedman's repeated measures analysis of variance by ranks, with Bonferroni‐Dunn's correction, was used to estimate the significance levels of the observed differences between the analyzed time points during the whole sporting season. Potential associations between changes in cortisol and testosterone with physical parameters (40 m, YYIRTL1 and VO_2max_) were tested using the Spearman correlation coefficient (*r*). *p* < 0.05 was accepted as a level of statistical significance.

## 3 RESULTS

### 3.1 Anthropometric characteristics

Anthropometric characteristics (body mass index, height, weight, and % body fat) of football referees and control subjects determined before the beginning (T0) and at the end of the season (T3) (mean ± *SD*) are shown in Table 1.

### 3.2 Physical fitness characteristics

Before training (T0), the average resting HR was 66 ± 2 beats/min. Exercise training program executed by a football referee induced a reduction of resting HR, which in T1 decreased by 8.5% compared to T0 (Table 1). HR values measured in T2 and T3 were not different from T1. No differences in maximal and minimal basal arterial pressure were observed throughout the observation period (Table 1).

**FIGURE 2:**
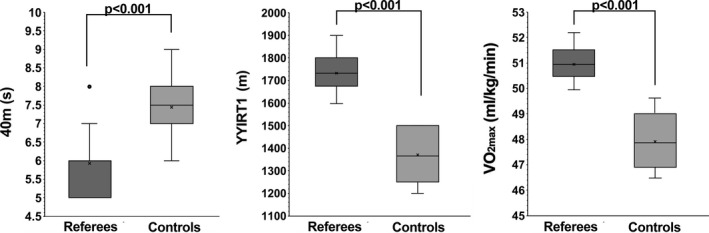
Physical parameter in football referees and controls before the beginning of the training period (T0, week 0). Box and Whiskers’ representation of running speed test, Yo–Yo intermittent recovery test level 1 (YYIRT1) and VO_2max_ values in referees (*n* = 29) and control subjects (*n* = 30). *p*‐values were obtained by Student's unpaired *t*‐test. In this representation, the central box covers the middle 50% of the data values, between the upper and lower quartiles. The bars extend out to the extremes, while the central line is at the median. Those values which are beyond 1.5 times the interquartile range beyond the central box are plotted as individual points

Figure 2 shows a Box and Whisker Plot of physical parameters before the beginning (T0) of the training for referees compared to control subjects. As can be seen, all the parameters (40‐m Sprint test, YYIRT1 and VO_2max_) referred to the referees were significantly different from the control subjects, demonstrating better physical performances.

**FIGURE 3:**
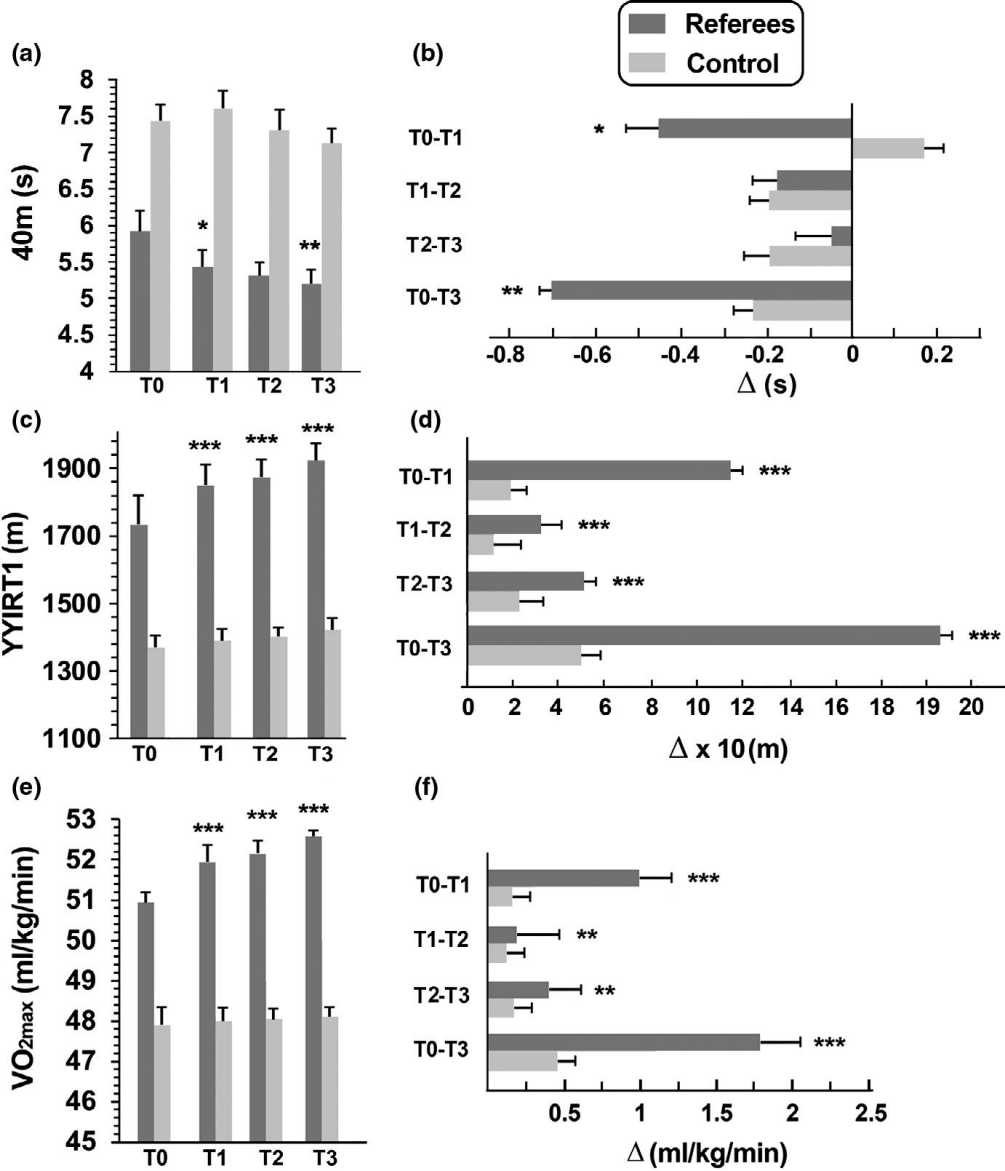
Physical parameter in football referees and their evolutions during a football season follow‐up. Running speed test (a,b), Yo–Yo intermittent recovery test level 1 (YYIRT1, c,d), and maximal oxygen consumption (VO_2max_) (e,f). The football referees were evaluated at four different time points, as in Figure 1. (a,c,e) The data are presented as mean ± *SD* and significant differences between groups were evaluated by ANOVA test. (b, d, f) The differences (Δ) of running speed tests (b), YYIRT1 (d), and VO_2max_ (f), between each time point, during a football season follow‐up. The data are presented as mean ± *SD*. Significant differences between time points were assessed by ANOVA followed by the Bonferroni test. Significant differences between T0–T1, T1–T2 and T2–T3, and T0–T3 were evaluated by *t*‐test. *p < 0.05; **p < 0.001, ***p < 0.0001

The changes in physical parameters were then evaluated at four different time points: before the beginning of the training period (T0), at the start of the pre‐season (T1), at the middle of the season (T2), and at the end of the season (T3). Football referee performances improved, while no significant differences were found at the same time point in control subjects (Figure 3). Precisely, at T0, the mean (±*SD*) for the 40 m Sprint test was 5.93 ± 0.37 s. Significant differences (*p* < 0.05) were noted for 40 m Sprint test in T2 and T3 (Figure 3a,b). At T0, the mean (± SD) for YYIRT1 was 1732.3 ± 105 m. The mean distance covered by the referees during the YYIRT1 increased significantly from T0 to T1, T2, and T3, and from T1 to T2 and T2 to T3 (Figure 3c,d). VO_2max_ also significantly increased during the season (Figure 3e) and significant differences were also found for VO_2max_ in T0‐T1, T1‐T2, T2‐T3, and T0‐T3 (Figure 3a,f).

### 3.3 Hormonal assays

**FIGURE 4:**
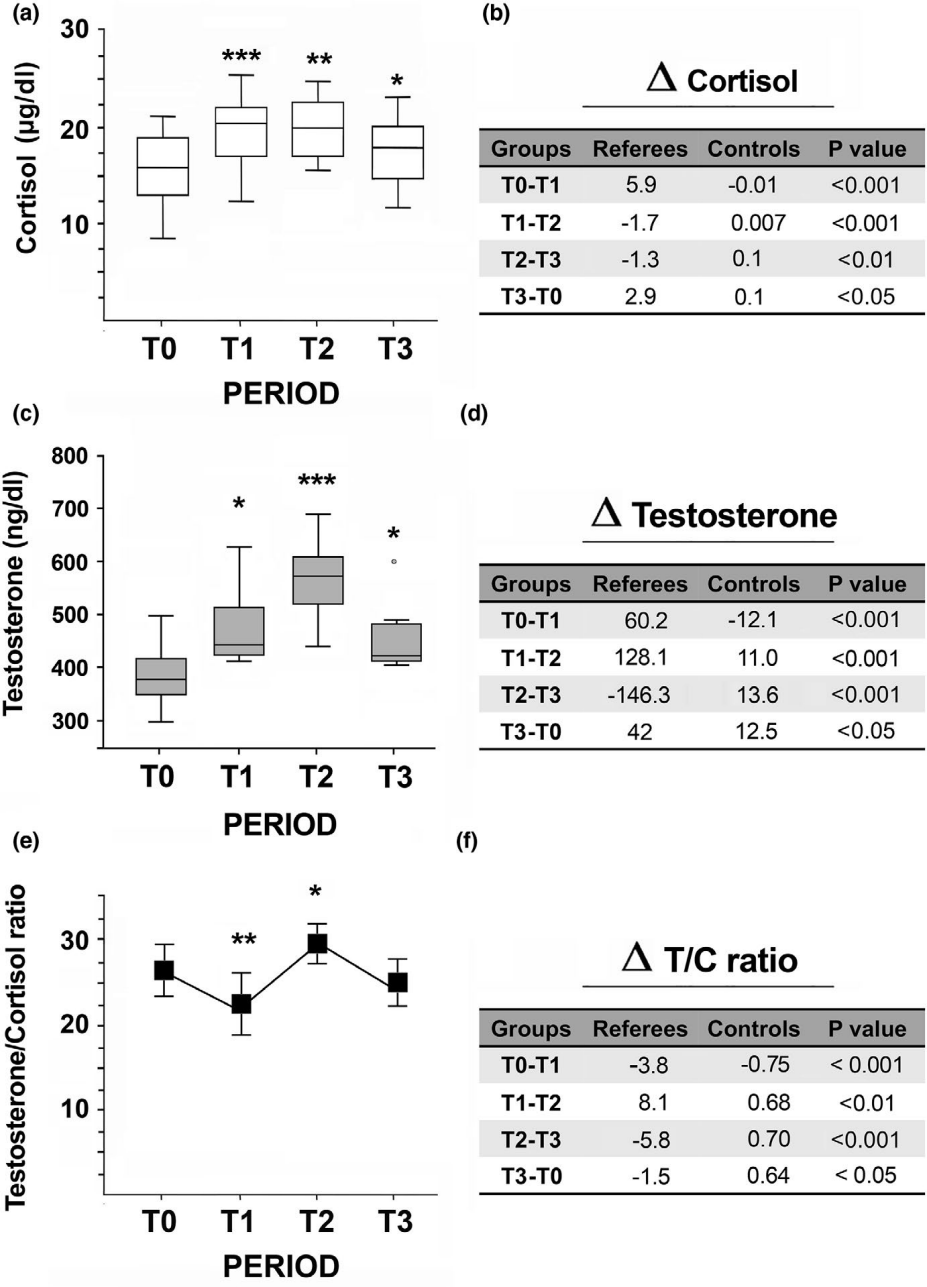
The effects of training on serum cortisol and testosterone in football referees. Total serum cortisol (a) and testosterone (c) concentrations were analyzed at each point‐time during the football seasons’ follow‐up. In this representation, the central box covers the middle 50% of the data values, between the upper and lower quartiles. The bars extend out to the extremes, while the central line is at the median. *p < 0.05; **p < 0.01; ***p < 0.001 by ANOVA followed by Bonferroni test between each point‐time and T0. (e) Testosterone to cortisol ratios were analyzed each point‐time during the soccer seasons follow‐up (mean ± *SD*). (b, d, f) Tables show the differences (Δ) in cortisol (b) testosterone (d) concentrations and testosterone to cortisol ratios (f) between each point‐time in football referees compared with control subjects. *p* values obtained by *t*‐test show differences in Δ values between each point‐time in referees compared with control subjects

In football referees, the cortisol concentrations increased during the first training period (T0–T1: from 15.8 ± 3.8 µg/dl to 21.7 ± 5.1 µg/dl, *p* < 0.001), then, slightly decreased during mid‐season (T2: 20.0 ± 1.9 µg/dl) and, at the end of the season, the concentration reached about initial values (T3: 18.7 ± 2.4 µg/dl) (Figure 4a). No significant differences were found in control subjects (Figure 4b).

Before the beginning of the training period (T0), the mean (±*SD*) testosterone concentration was 386.1 ± 58.8 ng/dl (Figure 4c); then it increased in T1 (444.2 ± 72.1 ng/dl; *p* < 0.05) and T2 (572.2 ± 88.1 ng/dl; *p* < 0.001), while at the end of the season, the concentration decreased toward initial levels (T3: 425.7 ± 78.2 ng/dl, *p* < 0.05) (Figure 4c). Testosterone to cortisol ratio also showed significant changes along the season. It decreased just after the training period (T1: *p* < 0.01), reached high levels at the middle of the season (T2: *p* < 0.05), and then returned to initial levels at the end of the season (T3: *p* > 0.05, Figure 4e).

Serum cortisol and testosterone concentrations were also analyzed in control subjects and no significant differences were found between each point‐time. Figure 4b,d show variations in hormone concentrations in referees and control group. *p* values show differences in cortisol and testosterone concentrations and testosterone to cortisol ratios between each point‐time in football referees compared to control subjects (Figure 4f).

### 3.4 Correlations between hormone concentrations and physical performances

**FIGURE 5:**
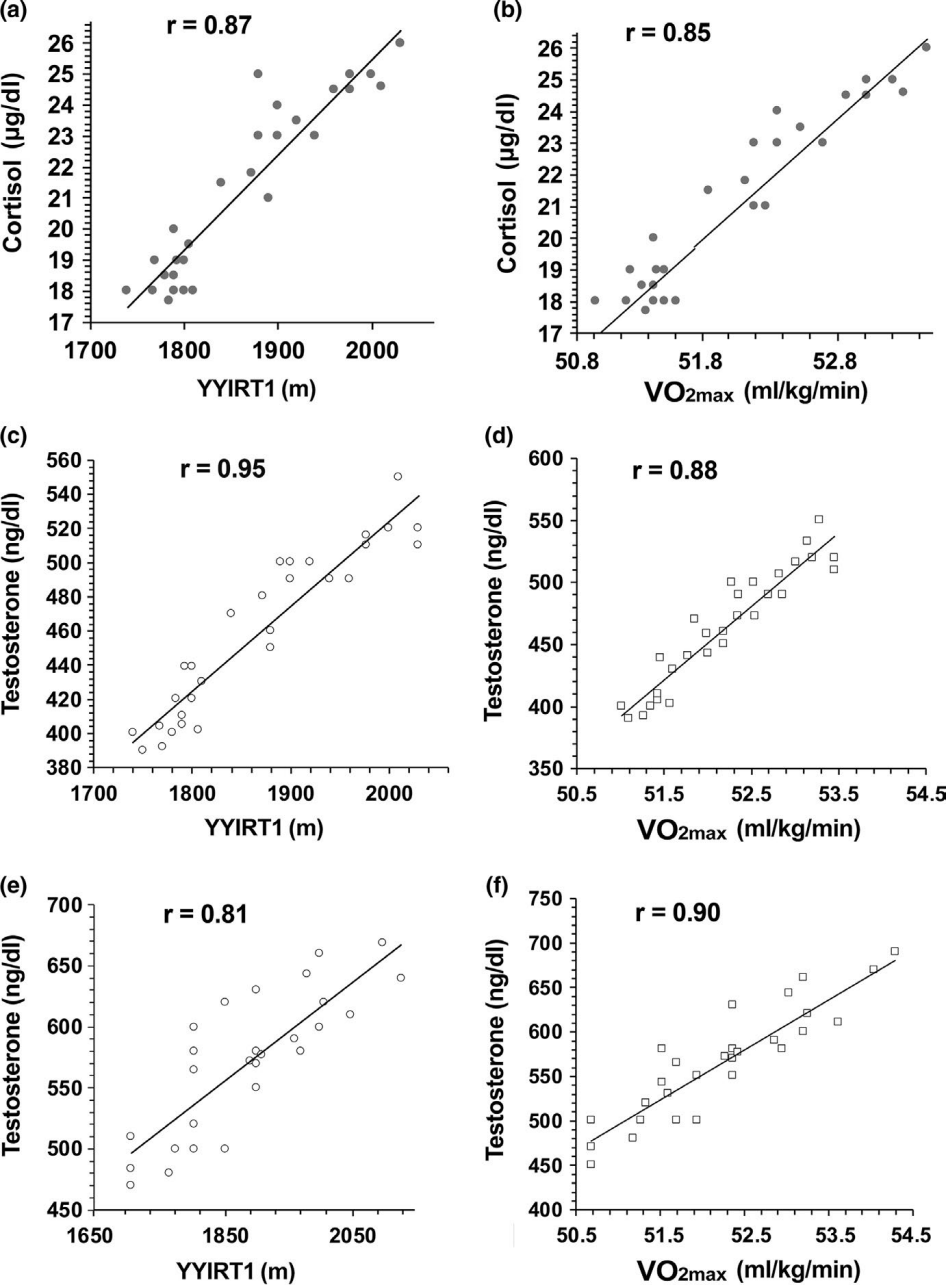
Correlations between cortisol and testosterone changes and physical performance. Scatter plots of cortisol concentration values and YYRT1 (a) and VO_2max_ (b) with linear regression at the end of the training period (T1). *p*‐values obtained by Spearman's rank correlation were *p* < 0.0001. Scatter plots of testosterone concentration values and YYRT1 (c) and VO_2max_ (d) with linear regressions at the end of the training period (T1). In addition to the correlations in T1, testosterone was also quantitatively associated with YYRT1 (e) and VO_2max_ (f) in the T2 period. *p*‐values obtained by Spearman's rank correlation were *p* < 0.0001

After the training period (T1), both cortisol and testosterone plasmatic concentrations were significantly correlated with YYIRT1 and with VO_2max_ (*p* < 0.0001 for all by Spearman's rank correlation, Figure 5a–d). At the middle of the season (T2), plasma testosterone concentrations only correlated with YYIRT1 and VO_2max_ (*p* < 0.0001 for both by Spearman's rank correlation, Figure 5e,f).

Since these correlations were not observed in control subjects, the exercise intensity influences the response of the hypothalamic–pituitary–adrenal axis. Collectively, these findings support the view that moderate to high ‐intensity exercise increases circulating cortisol and testosterone levels.

## 4 DISCUSSION

Football referees must be able to withstand playing time in order to make appropriate decisions (Weston et al., 2012). Consequently, at the beginning of the competitive season, the governing boards request the evaluation of the physical form of the referees, also setting the limits for inclusion in the list of all‐season match assignments. Hence, football referees need adequate and specific physical preparation to be able to carry out all the assigned matches. Physical training protocols are effective when they induce all the adaptations that can determine the evident improvement in sports performance. Hence, the need for a better understanding of the referee's fitness progress in order to properly adapt their training protocols. Various football‐related factors, such as fixture time, tactics, environmental conditions, championship competition and, above all, the training, can influence endocrine physiological responses (Aldous et al., 2016; Arruda et al., 2015; Hammami et al., 2017). Consequently, the physiological response of hypothalamic–pituitary–adrenal axis to training and competition has been seen within football (Slimani et al., 2017). However, few studies have investigated cortisol and testosterone concentration changes in referees during a professional football match (Kokaly et al., 2018). Furthermore, studies examining the effects of specific training programs able to improve the physical performance of football referees are very few. We hypothesized that high‐level training programs formulated by the technical area, the athletic preparation section of the “Associazione Italiana Arbitri”, and match stress would be accompanied by hormonal changes promoting physiological adaptations and tested it in Italian football referees. During a match, the football referee runs many meters both in the first and in the second time (D'Ottavio & Castagna, 2001) and physical activity is characterized by frequent periods of intense exercise alternating with low‐intensity periods (Krustrup et al., 2002). In this report, we demonstrate that during the football season, and following the training protocol, the referees improved their aerobic and anaerobic performance, as demonstrated by YYIRT1. Indeed, YYIRT1 is correlated with the referees’ performance (Krustrup & Bangsbo, 2001), since during this test the anaerobic system is heavily loaded, and the aerobic load is close to the maximum values (Muscella et al., 2020). At the end of the competitive season, and despite the individual differences, the referees improved their fitness which becomes comparable to that of the soccer players (Krustrup et al., 2003; Muscella et al., 2019). In addition to aerobic fitness, repeated sprint performance is another crucial element for athletes (Girard et al., 2011; Ruscello et al., 2013). The fastest 40‐m time sprint test had appropriate construct validity for the physical assessment of football referees (Weston et al., 2009). Our results showed that performance improved mainly between T0 and the end of the season. Conflicting results are reported in the literature regarding the relationship between the sprint test and both aerobic and anaerobic indices. In several studies, higher VO_2max_ appears to be related to better sprint test performance (Jones et al., 2013; Rampinini et al., 2009), allowing the replenishment of phosphocreatine reserves during recovery between sprints, thus maintaining high‐intensity performance (Bogdanis et al., 1996). Indeed, the training sessions to which the football referees were subjected resulted in good levels of aerobic and anaerobic fitness. These results could also be extended to referees from other sports, as insufficient fitness levels could compromise the referees’ ability to sustain the pace of the game and therefore to correctly arbitrate. Football referee fitness levels are correlated with match performance (Krustrup & Bangsbo, 2001). Not surprisingly, the progress in referees’ physical fitness has a significant impact on match performance; this also has important effects on the selection of the referees by the governing bodies.

Various studies have examined match activities and fitness characteristics of football referees (Castagna & D’Ottavio, 2001; Catterall et al., 1993; D'Ottavio & Castagna, 2001; Tessitore et al., 2007; Weston et al., 2007). These studies have examined the distance and intensity of movements (Castagna & D’Ottavio, 2001; D'Ottavio & Castagna, 2001; Weston et al., 2007), the heart rate (HR) profile (Catterall et al., 1993; Tessitore et al., 2007; Weston et al., 2006), and the effect of a soccer match on the cardiac autonomic control of HR (Boullosa et al., 2012) in order to assist in the referees’ preparation. Nevertheless, our study is the first to monitor hormones and the longitudinal effect of a training load stimulus (one season follow‐up) in football referees. Circulating cortisol level controls catabolic adaptations to exercise training and relies on physical fitness level, duration and intensity of exercise, nutritional status, and circadian rhythm. In addition, cortisol may also be considered as an indicator of excessive stress and over‐reaching/over‐training in athletes (Hammami et al., 2017; Kraemer et al., 2004) with associated performance decrements. In line with our previous study performed in young football players (Muscella et al., 2019), in this study we observed changes in plasma cortisol during the follow‐up period in football referees possibly related to physical stress, with an increment by 37% during T0–T1 point‐time. The effects of sport on cortisol blood concentrations are not yet defined. Specifically, some authors showed no changes in resting cortisol values (Filaire et al., 2001), others reported a concentration decrement (Bonifazi et al., 2000), and still, others associated the best performance in football, especially in very intensive training programs, with a significant increase in plasma cortisol concentrations (Bonifazi et al., 2001). Currently, there is only one study showing a 48% increment in salivary cortisol levels in 16 elite young male referees after a single football match (Kokaly et al., 2018). As for testosterone, numerous studies have shown its effect in mediating adaptive training responses (Crewther et al., 2006). In our study, we observed that after an intense training period, the testosterone concentration increased by 32% and then returned to baseline levels at the end of the season. This may suggest that the increase in physical performance may also depend on the increase in plasma testosterone levels associated with the decrease in cortisol. The training‐induced increases in serum testosterone levels reflect the increase in anabolic activity (Kraemer & Ratamess, 2005). However, these hormone concentration changes cannot be considered clinically relevant to health because values remained in normal ranges, as reported also by several other studies (Hammami et al., 2017; Hansen et al., 1985; Makras et al., 2005; Muscella et al., 2019). The variations observed between the periods T0 and T2 can represent a typical homeostatic modulation responding to adaptive processes to training, to the stressful environment, and to competitions (Muscella et al., 2019; Young et al., 2012). Thus, results suggest that training leads to improvement of physical fitness performances and to plasma cortisol and testosterone concentrations increment, compared to control subjects, perhaps representing an adaptation to exercise.

The role of the testosterone/cortisol ratio is controversial (Cadegiani & Kater, 2017). However, it may indicate a disproportion between catabolic and anabolic metabolism and stress due to training (Adlercreutz et al., 1986). Therefore, expressing the balance between catabolic and anabolic processes could be useful in assessing the impact of training and competition (Urhausen et al., 1995). From this point of view, a ratio showing high concentrations of cortisol may indicate overtraining, thus suggesting the opportunity to modify the referees’ training schedule during the football season. We here found that, compared to the initial period T0, this ratio decreased in T1 by 15%, detecting a modest overtraining; then T/C ratio increased by 16% in T2, perhaps due to an adaptive recovery, and finally, decreased modestly (by 5.5% with respect to T0) at the end of the season (T3) thus showing a trend to recover optimal conditions. In control subjects, this ratio did not undergo any significant variation. We may assume that referees have not been overtrained and adequately respond to coaching without accumulating fatigue.

The variation in cortisol and testosterone levels strongly correlated with fitness performances (YYIRT1, VO_2max_); thus, such variations may be considered as an endocrine marker of physical fitness in football referees. However, some limitations concerning our experimental protocol should be taken into account. For instance, blood samples were taken only at rest; since referees are subjected to physical but also mental stress due to complex decision making by dealing with players, coaches, and audiences, it would be interesting to take blood samples during, at half time and at the end of the match in order to identify the cortisol role in the regulation of physical and mental stress. In addition, the effect of other variables such as diet, sleep, or motivation, and psychological behavior (stress, anxiety, or sleep quality) should be addressed in the upcoming studies, because these factors influence the hormonal responses. Finally, another limitation of our study is the small sample size. Nevertheless, results in terms of changes in hormone concentrations and their relationship to physical performance were observed in the referee group but not in the control group of 30 non‐athletic men. However, further studies are desirable to increase the number of cases in order to consolidate these data.

Referee training leads to cortisol and testosterone changes in order to promote physiological adaptations. Hence, the training load of the football referees can be suitably balanced and modulated following the specific hormonal response in order to achieve two fundamental objectives: the improvement of physical performance and overtraining avoidance.

## COMPETING INTERESTS

The authors declare that they have no competing interests and that they have ethics approval and consent to participate.

## CONSENT FOR PUBLICATION

The authors give the consent for publication and their availability of data and material.

## AUTHOR CONTRIBUTIONS

A.M. and S.M. designed the study. E.S. collected and provided physical fitness and analyzed the data. A.M. and S.M. generated the figures and cowrote the paper. All authors read and approved the final manuscript. The manuscript has not been published or submitted for publication elsewhere.
